# The nudging effect of AIGC labeling on users’ perceptions of automated news: evidence from EEG

**DOI:** 10.3389/fpsyg.2023.1277829

**Published:** 2023-12-22

**Authors:** Yuhan Liu, Shuining Wang, Guoming Yu

**Affiliations:** ^1^School of Journalism and Communication, Beijing Normal University, Beijing, China; ^2^Laboratory of Cognitive Neuroscience and Communication, School of Journalism and Communication, Beijing Normal University, Beijing, China; ^3^State Key Laboratory of Media Convergence Production Technology and Systems, Beijing, China

**Keywords:** automated news, users’ perception, AIGC labeling, nudge theory, EEG

## Abstract

**Introduction:**

In the context of generative AI intervention in news production, this study primarily focuses on the impact of AI-generated content (AIGC) labeling cues on users’ perceptions of automated news based on nudge theory.

**Methods:**

A 2 (authorship disclosure nudge cues: with vs. without AIGC label) × 2 (automated news type: descriptive vs. evaluative news) within-subject experiment was carried out. Thirty-two participants were recruited to read automated news, evaluate the perceived content trustworthiness, and record with an EEG device.

**Results:**

The results demonstrated that disclosure of AIGC labeling significantly reduced the trustworthiness perception of both fact-based descriptive and opinion-based evaluative news. In EEG, the delta PSD, theta PSD, alpha PSD, and beta PSD with disclosure of AIGC labeling were significantly higher than those without AIGC labeling. Meanwhile, in descriptive news conditions, TAR with AIGC labeling was higher than without AIGC labeling.

**Discussion:**

These results suggested that AIGC labeling significantly improves the degree of attention concentration in reading and deepens the degree of cognitive processing. Users are nudged by AIGC labeling to shift their limited attention and cognitive resources to re-evaluate the information quality to obtain more prudent judgment results. This helps to supplement the theoretical perspective on transparent disclosure nudging in the Internet content governance research field, and it can offer practical guidance to use content labeling to regulate the media industry landscape in the face of AI’s pervasive presence.

## 1 Introduction

The introduction of large language models, such as ChatGPT, indicates that artificial intelligence (AI) has made significant strides in the field of content creation. Generative AI has been widely regarded as one of the most revolutionary and disruptive tools, penetrating previously unimaginable domains, such as painting, poetry writing, and professional news reporting (Carlson, 2017). Man–machine collaboration-produced automated news has progressively become the norm in the media industry, and news production has become more efficient, accessible, and cost-effective (Chen, 2023).

However, this highly automated production approach presents new obstacles. As generative AI evolves, it becomes more difficult to distinguish between machine-written and human-written texts, whether in simple texts or complex texts containing multimodal and emotional elements (Wölker and Powell, 2018). Because generative AI shares the same journalistic ethical concerns, including black box algorithms, prejudice and bias, and vulgarity, the concealment of the technology accentuates these issues (Graefe et al., 2016; Dwivedi et al., 2023). These can result in more severe misinformation for news consumers. As the coding website Stack Overflow states, ChatGPT generates answers with a high error rate, but they are typically realistic and simple to generate (The Heart of Machine, 2022). NewsGuard tested ChatGPT and found that it generated error messages 80% of the time and presented them more convincingly (NewsGuard, 2023). Other scholars have referred to this low-quality, indistinguishable AI-generated content as *AI hallucinations*, in which machines express themselves convincingly but in a completely fabricated way (Edwards, 2023). This opaque AI technology logic is invading the media industry, blurring users’ judgment about news quality, and possibly even diminishing the credibility of news organizations. When users regard news organizations as untrustworthy providers of information on major events, the entire social fabric will be shattered (Schulhofer-Wohl and Garrido, 2012).

In the media industry, the paradigm of automated news production with generative AI highlights the importance of transparency disclosure. Transparency disclosure involves making the process of gathering, organizing, and spreading information open, which allows both inside and outside parties to monitor, investigate, criticize, and even intervene in news production (Deuze, 2003). Attributes seen in more ubiquitous examples of transparency disclosure include providing information about story corrections, author biographies, and hyperlinks to related stories and documents (Chadha and Koliska, 2014). It moderates user perception and improves the news industry’s overall credibility. These proactive disclosure approaches that improve information accuracy and clarity would become a form of soft content governance, which can assist users in processing information in a non-misleading manner and promote a healthier and more organized communication ecology through small nudging cues (Lorenz-Spreen et al., 2020; Pennycook and Rand, 2022). Specifically, the researchers noted that the authorship disclosure of automated news was a crucial component of the transparent disclosure nudge, which can inspire users’ sociocultural perceptions about AI as a news producer and affect users’ cognitive evaluations (Reich and Klein-Avraham, 2014; Montal and Reich, 2016). The theory of interactive media effects (TIME) model proposed by Sundar et al. further illustrated that users’ evaluations of human-authored and machine-authored works differed drastically. Interface machine cues activate users’ machine heuristic thinking paths, which can shape users’ content perceptions and even the entire user experience (Sundar et al., 2015). Empirical research found that the effect of influencing users’ perceptions of authority and credibility can be accomplished through the modest means of bylines for AI creators (Das and Pavlíčková, 2013; Dwivedi et al., 2023). The Chinese short video platform *DOUYIN* has introduced AI-generated content (AIGC) labeling to disclose news authorship, implying that the information may have been autonomously generated by AI (Tencent News, 2023). Morrow et al. (2022) emphasized that in future, supplying content labeling, such as AIGC labeling and fact-checking labeling, will be an important content governance tool in human–machine collaborative journalism. The literature on automated news is replete with comparative studies on human and machine news writing and reached the consensus conclusion that users perceive human and machine writing differently (Tandoc et al., 2020). However, few studies have considered authorship disclosure labeling in automated news, disregarding the significance of AI authorship disclosure in user cognitive evaluations. To date, no study has examined the potential impact of AIGC labeling on users’ news exposure.

Given this, the present study will concentrate on the transparent cues of AI authorship, utilizing EEG technology and behavioral experiments to determine whether AIGC labeling affects users’ perceptions of automated news. In light of the possibility that the heuristic reasoning of users influenced by AIGC labeling may differ between descriptive and evaluative news (Longoni and Cian, 2020), the present study will also investigate the potential interactive effects of news type. Theoretically, the current study can supplement the theoretical perspective on transparent disclosure nudging in the Internet content governance research field and confirm the efficacy of transparent disclosure via authorship labeling cues. In a practical sense, the present study can offer media organizations guidance on how to utilize AIGC labeling and provide a view of how content is governed by content labeling, thereby helping to regulate and direct the media industry landscape in the face of AI’s pervasive presence.

## 2 Literature review

### 2.1 Nudge theory and transparency disclosure nudge

In their 2008 book *Nudge: How to Make the Best Decisions About Health, Wealth, and Happiness*, behavioral economist Richard Taylor and jurist Cass Sunstein proposed nudge as an independent theoretical concept. Nudge involves designing and implementing choice situations via symbols and innovations with the intent to alter people’s behaviors in predictable ways without significantly changing incentives for conformity or overt, punitive repercussions for non-conformity (Thaler and Sunstein, 2008). Simply stated, nudging is an indirect method of interfering with users’ decision-making behaviors that minimizes resistance to intervention while preserving the user initiative. Sunstein (2021) further suggested that nudges fall into two categories: educative and architectural. Educative nudges include warnings, reminders, and disclosure of information (such as calorie labels, allergy warnings, and fuel economy labels). Architectural nudges include automatic enrolment, mandatory choice, simplification, and sludge reduction. From the perspective of human information processing patterns, the psychological mechanism of nudging works can be traced back to at least Simon’s research on bounded rationality (Simon, 1955) and Daniel Kahneman and Amos Tvisky’s research on cognitive operations (Kahneman and Tversky, 1972). Within behavioral science, some people have found it helpful to distinguish between two families of cognitive operations in the human mind: *System I*, which is fast, automatic, and intuitive; and *System II*, which is slow, calculative, and deliberative. Educative nudges attempted to strengthen the hand of *System II* by improving the role of deliberation and people’s considered judgments. Architectural nudges were designed to appeal to, or to activate, *System I*. In contrast to architectural nudges, educative nudges aimed not only to preserve freedom of choice but also to increase individual agency. Consequently, scholars supported that educative nudges overlapped with short-term boosts, which focused on not only design choice architecture but also design choice information to make people think more rationally. Both represent local fixes to a given problem and require—in contrast to classic architectural nudges, such as defaults—a modicum of motivation and cognitive skill (Hertwig and Grüne-Yanoff, 2017). The benefits of educative nudges include the following: First, microdesign modifies the provided information through minor cues; and second, mild persuasion intervenes with individuals based on the principle of preserving users’ freedom and increasing users’ agency. In the news communication context, users tend to process information with as little cognitive effort as possible. For instance, most users do not exert much effort to carefully peruse the news. Instead, they understand the information by glancing at the information title and other peripheral factors (Weinreich et al., 2008). Researchers have found that labels or alerts often function as educative nudges to help users think critically (Lorenz-Spreen et al., 2020; Pennycook and Rand, 2022).

Theoretically, transparent disclosure may be an effective educative nudge for influencing users’ cognitive processes. Transparent cues convey the information production process to users, highlighting the degree of subjectivity and potential quality risks that may exist in the information and nudging users to refocus attention on the quality of the information. The so-called transparency cues are all informational elements that can demonstrate the transparency of the news production process, including a detailed description of the news source (i.e., supplements to the news source and statements of authorship) (Reich and Klein-Avraham, 2014), and elaborate explanations of the news-gathering process (Rupar, 2006). The study by Newman and Fletcher (2017) summarized several important transparency cues that media organizations must disclose, including interview statements, authorship disclosures, content subjectivity notices, references, and editorial statements. In automated news, the authorship disclosure serves as a machine–creator authorship disclosure and an explanation of the algorithm model. Multiple studies have confirmed the nudging effect of transparent disclosure cues in the context of human-written news consumption. A study found that embedding reference hyperlink cues and providing information correction cues can significantly improve users’ perceived trustworthiness (Chadha and Koliska, 2014). Disclosing authors’ resumes and details can not only improve the trustworthiness evaluation of news content but also enhance users’ news-sharing willingness (Curry and Stroud, 2019). In addition, another study found that including an opinion label (indicating that the article includes subjective opinion expression) can significantly increase users’ perceived trustworthiness of news sources (Otis, 2022).

In light of this, the present study infers that a similar educative nudging effect should be expected from authorship disclosure cues in the automated news exposure process. Therefore, the following is hypothesized:

*H1*: AIGC labeling affects users’ perceptions of the trustworthiness of automated news content.

### 2.2 The impact of authorship disclosure on content trustworthiness

Because it entails a declaration of the information source, authorship disclosure is a crucial aspect of transparency disclosure. The significance of information sources in content evaluation is self-evident. When an information source is thought to be of high quality (Chaiken, 1980), consumers frequently believe that the information delivered by it is also of high quality. Existing research has demonstrated that authorship as an information source cue has the potential to influence information reliability judgment. Choi and Lim (2019) discovered, for example, that when online news reporters were verified, people viewed the content as more trustworthy. Krouwer et al. (2019) discovered that stating which portion of news works is produced by marketers and incorporates advertising sponsorship and which portion is produced by journalists can greatly improve users’ perceived transparency and credibility of the materials. In automated news, the disclosure of machine authorship is more significant than the disclosure of human authorship. As a means of recognizing the creativity of machines and the subjectivity of copyright, bylining AI has been argued by numerous legal, literary, and philosophical scholars (Davies, 2011). Currently, the method for revealing machine authorship via byline is not universally accepted. As an alternative, straightforward labeling is used to declare machine authorship to alert users. These labels are usually phrased as *this content was generated by AI*. The current study refers to such labels as AIGC labels.

In the past, many studies have confirmed that users hold different reliability evaluations for information from two distinct sources (Liu and Wei, 2018; Tandoc et al., 2020), humans and machines. Whether to trust machine writing or human writing more, the findings, however, are somewhat mixed. In general, machine-written news is perceived to be less emotionally engaging in fact-based news conditions, so it may be evaluated as more credible (Liu and Wei, 2018). However, machine-written opinion-based explanatory news is perceived to lack professionalism and depth and is evaluated as less credible. Tandoc et al. (2020) compared automated earthquake news based on objectivity and evaluative writing and discovered that the trustworthiness of AI-generated content decreased for evaluative texts, whereas there was no difference between objective and evaluative writing styles in human-generated content. Similar to the findings of Castelo et al. (2019), when content production entails intuition, emotion, or empathy, AI author ratings decrease. These results supported the machine heuristic model proposed by Sundar and Kim (2019), which contends that when AI-related cues are present, people are motivated to take mental shortcuts to process information. It encompasses both positive and negative machine heuristic paths. The positive machine heuristic path acknowledges the more objective and accurate content production capabilities of machines than those of humans, which may increase individuals’ information evaluations. The negative machine heuristics path queries the ability of machines to make subjective judgments, use interpretive reasoning, and have emotional empathy in comparison with human creators, which may diminish individuals’ information evaluations. Longoni and Cian (2020) named the possible evaluation effect of machine authorship as *the word-of-machine effect*, in which AI is perceived to be more competent for utilitarian realms and functional goals than for hedonistic realms and affective goals. Therefore, the type of news can be considered the most important interactive variable for the nudging effect of machine authorship disclosure. That is, the cognitive consequences for users nudged by AIGC labeling may depend on the type of automated news. When automated news is factual news based on statements and descriptions, AIGC labeling may nudge the user to take a positive machine heuristic path, whereas when automated news is opinion-based news, AIGC labeling may nudge users to take a negative machine heuristic path.

Nevertheless, the literature on automated news evaluation has two deficiencies. First, the majority of studies primarily focused on exploring the effect of authorship itself but not the effect of authorship disclosure. For example, studies often investigated two types of content, human-generated and machine-generated contents, which means there was a creator difference between texts (Liu and Wei, 2018). The current study focused on the impact of authorship disclosure, which means providing notices of automated news, and explored the potential effect of declaring machine authorship on machine-generated content as a text type. Second, the few studies on machine authorship disclosure are too old to accurately reflect the interactive relationship between humans and AI today. On the one hand, the majority of AI technologies utilized in these studies were more backward discriminative AI rather than generative AI (Discriminative AI trains models with labeled data, guiding the models to acquire the ability to give answers to questions. Generative AI trains models with unlabeled data and self-supervised learning, guiding the models to generate contextualized content. Generative AI outperforms discriminative AI in process explanation and emotional expression). The efficacy of machine-generated content in complex texts, such as emotional and interpretational content, is poor. Users can differentiate between human- and machine-generated contents to a high degree. On the other hand, as AI technology advances, users become more psychologically receptive to the machine coproduction mode of life, which means that their AI rejection and algorithmic aversion emotions may be attenuated (Dietvorst et al., 2015). Thus, there may be changes in the effect of machine cues to influence people’s information evaluations.

Based on the theoretical derivation of transparent disclosure nudges, the present study plans to investigate the effect of AI authorship disclosure (i.e., the presentation of AIGC labeling) on user perceptions of automated news in the context of generative AI. In addition, in conjunction with the positive and negative machine heuristic models proposed by Sundar and Kim, the current study considered the possible interaction effect of automated news types in the nudge effect of AIGC labeling, focusing on two news types: descriptive news based on factual description and evaluative news based on subjective opinion expression. In this study, descriptive news was defined as a type of text that explicitly presents the elements of objective news facts and avoids subjective evaluation, and evaluative news was defined as a type of text that demonstrates subjective views and reduces the language of factual description. In summary, the following hypotheses are formulated:

*H2*: In descriptive automated news, disclosing AIGC labeling nudges users to increase their perceived trustworthiness compared to not disclosing AIGC labeling.

*H3*: In evaluative automated news, disclosing AIGC labeling nudges users to reduce their perceived trustworthiness compared to not disclosing AIGC labeling.

### 2.3 The nudging effect of AI authorship disclosure in the cognitive-physiological dimension

As mentioned above, previous studies on machine authorship disclosure lacked more in-depth cognitive research tools, and its impact was mainly measured through self-reported methods, such as questionnaires or behavioral experiments. However, these measurements are always challenged by subjectivity and lack of granularity, as people do not always know what they need. As Steven Quartz, a cognitive neuroscientist at Stanford University, has pointed out, no matter how objective a questionnaire’s results may be, it is still a judgment made by the brain after postprocessing, and in fact, much of the demand comes from the preprocessing of information (Yu, 2018).

Therefore, in recent years, many researchers (not the least of whom are communication scholars) have begun to use cognitive neural measurement tools, such as electroencephalography (EEG), eye movement, and functional magnetic resonance imaging (fMRI), to measure users’ microprocesses of information processing and explain communication problems by measuring individual physiological indexes. Some consumer neuroscience or neuromarketing researchers have noted that exogenous cues, such as brand labeling in marketing, can cause different physiological responses in consumers, and EEG measurement tools are the most appropriate and important tools to explore users’ cognitive processes (Clark et al., 2018). This is because EEG can carefully and objectively record and respond to individuals’ cognitive engagement and mental workloads, which can reveal individuals’ implicit and real-time cognitive performance. EEG is a non-invasive brain imaging method that uses different electrodes placed on the scalp to detect electrical activity in the brain, recording and storing an individual’s cognitive activity throughout the cognitive process. At present, some studies have explained the effects of media on physiological levels by combining EEG measurement methods. For example, Han et al. (2020) used EEG equipment to collect ERP data, such as late positive component (LPC), to compare the effects of paper media and electronic media on the brain mechanism of users’ information processing. Xiu et al. (2023) illustrated the impact of conversational news on cognitive absorption and user experience by describing the performance of the theta band. To analyze EEG data, researchers often use the power spectral density (PSD) approach. PSD can measure the frequency distribution of power in EEG data and extract signal features from four EEG bands (delta band, theta band, alpha band, and beta band), which have been proven to be strongly associated with human brain information processing processes. They were useful indices for studying media effects on the cognitive-physiological level. Traditionally, the delta band was regarded as the primary indicator of cognitive fatigue (Lal and Craig, 2001). The theta band reflected the degree of distraction inhibition (Gruzelier, 2014). The alpha band represented the degree of cognitive arousal on the one hand and mental agility on the other (Borghini et al., 2014). The beta band showed attentional concentration (Fernández et al., 1995). Furthermore, EEG could also reflect metacognitive management processes by some indices, such as the theta/alpha ratio (TAR), theta/beta ratio (TBR), and frontal EEG asymmetry (FEA). FEA revealed the implicit motivation of information processing (Coan and Allen, 2004), and TAR and TBR revealed the degree of cognitive loading and cognitive control (Yu et al., 2021).

However, there is a gap in research on brain activity triggered by content labels (especially authorship labels). In combination with the research purpose, the present study attempted to utilize EEG measurement tools with the PSD analytical method and other EEG indexes (i.e., FEA, TAR, and TBR) to explore the nudge effect of AIGC labeling disclosure on users’ cognitions. Given this, the present study raises the following research questions:

RQ1: How will the disclosure of AIGC labeling affect cognitive brain activity in response to automated news?

## 3 Research method

In the current study, a 2 (authorship disclosure nudge cues: with AIGC label vs. without AIGC label) × 2 (automated news type: descriptive news vs. evaluative news) within-subject experiment was carried out. Different experimental conditions were presented to participants in the form of blocks. A total of four blocks were set up, each of which contained 20 experimental automated news materials. The order of block presentation was balanced in Latin squares among participants.

### 3.1 Participants

A 12-person pre-experiment was conducted, and the results showed that the effect size *f* of the possible labeling main effects was 0.42. G*Power 3.1 was used to calculate the sample size of the present study. The results showed that at least 20 participants were needed when the effect size was 0.42, and the statistical power was 0.8. A total of 36 participants were recruited in this experiment. All participants were undergraduates and postgraduates from universities in Beijing, China, with different disciplinary backgrounds. To avoid the interference of prior familiarity and ensure that all participants were seriously involved in automated news reading, a screening questionnaire of topic familiarity was used before the experiment; it consisted of two questions: *Have you heard of these topics before?* and *Have you seen these topics before?* All the questions were measured by a 7-level Likert scale. Participants who scored more than *±3 SD* on topic familiarity were excluded. After the experiment, a screening questionnaire for reading attention was also administered. Thirty reading detail test questions were adapted according to all the experimental automated news material, and the total score of correct answers was calculated. Participants whose total score was lower than the average score were excluded. Accordingly, two participants were excluded due to topic familiarity and two participants were excluded due to reading attention.

The final sample included a total of 32 participants (13 men and 19 women, average age 22.72 ± 2.48). All participants had normal or corrected-to-normal vision, were right-handed, had no current or history of neurological or psychiatric disorders, and did not take any psychoactive drugs, such as insomnia prescriptions or stimulants. Before the experiment, the Chinese versions of the Baker Anxiety Scale (BAI), Baker Depression Scale (BDI), and Positive and Negative Emotion Scale (PANAS) were used to assess the participants’ recent emotional status. The results were as follows: BAI 26.13 ± 4.67 points, BDI 6.91 ± 6.76 points, negative emotion 15.28 ± 5.77 points, and positive emotion 28.59 ± 8.07 points. None of the participants had significant clinical anxiety or depression symptoms. All participants signed informed consent forms and received cash rewards after the experiment.

### 3.2 Materials

#### 3.2.1 Automated news reading materials

Automated news reading materials included descriptive news and evaluative news. News topics were selected from the ranking lists of *JinriToutiao* (the largest news aggregation platform in China), covering current affairs, politics, social livelihood, economy, science and technology, entertainment and sports, etc. After collecting news topics in *JinriToutiao*, according to the experimental purpose, ChatGPT 4.0 was used to generate descriptive and evaluative news texts. The input command formats to ChatGPT 4.0 were as follows: (1) *Please write a piece of descriptive news about xxx (news topic), describing the event facts, including a 10-word title and an approximate 150-word body*. (2) *Please write a piece of evaluative news about xxx (news issue), presenting the opinions of events, including a 10-word title and an approximate 150-word body*. A total of 60 descriptive news and 60 evaluative news texts were generated. To ensure that the news generated by ChatGPT 4.0 was in line with the experimental manipulation and to exclude other possible material interference factors, 11 undergraduates with major backgrounds in journalism scored these 120 news materials in advance. They scored the degree of fact description, opinion commentary, and controversy. The degree of fact description was measured by two questions: (1) *To what extent does the content express the truth?* and (2) *To what extent does the content demonstrate objectivity?* The degree of opinion commentary was measured by two questions: (1) *To what extent does the content express opinion?* and (2) *To what extent does the content demonstrate subjectivity?* The controversy degree was measured by three questions: (1) *How accurate is the content?* (2) *How clear is the content?* and (3) *How reliable is the content?* All questions were measured by a 7-level Likert scale. In the end, news materials with controversy *scores outside ± 3 SD* were removed. Then, we selected the 40 news materials with the highest fact description scores as the final descriptive news reading materials, and we also selected the 40 news materials with the highest opinion commentary scores as the final evaluative news reading materials. In the final automated news reading materials, there were significantly different description scores between the descriptive news materials (*M* = 4.62, *SD* = 0.52) and evaluative news materials (*M* = 3.22, *SD* = 0.84) [*t*(10) = 3.979, *p* = 0.003, Cohen’s *d* = 2.00]. There were significant differences in opinion commentary scores between the descriptive news materials (*M* = 3.40, *SD* = 1.10) and evaluative news materials (*M* = 4.51, *SD* = 0.89) [*t*(10) = −3.067, *p* = 0.012, Cohen’s *d* = 1.11]. In terms of the controversy score, there was no significant difference between the descriptive news materials (*M* = 4.23, *SD* = 0.35) and evaluative news materials (*M* = 4.27, *SD* = 0.40) [*t*(10) = −0.290, *p* = 0.778, Cohen’s *d* = 0.11]. All the news materials were approximately 150 words long (*M* = 148.54, *SD* = 3.57).

#### 3.2.2 AIGC labeling

To manipulate the authorship disclosure nudge cues, some automated news materials in this study were labeled with AIGC labeling, while others were unlabeled. The AIGC labeling was designed by referring to the existing AIGC label cases of the *Douyin* platform and *Weibo* platform. AIGC labeling appeared in the format of a prompt bar between the news title and the news body, with white text on a gray background. It read *This content was generated by AI*. To eliminate distractions, AIGC labeling appeared randomly and evenly in all automated news reading materials.

### 3.3 Experiment procedure

**Figure 1:**
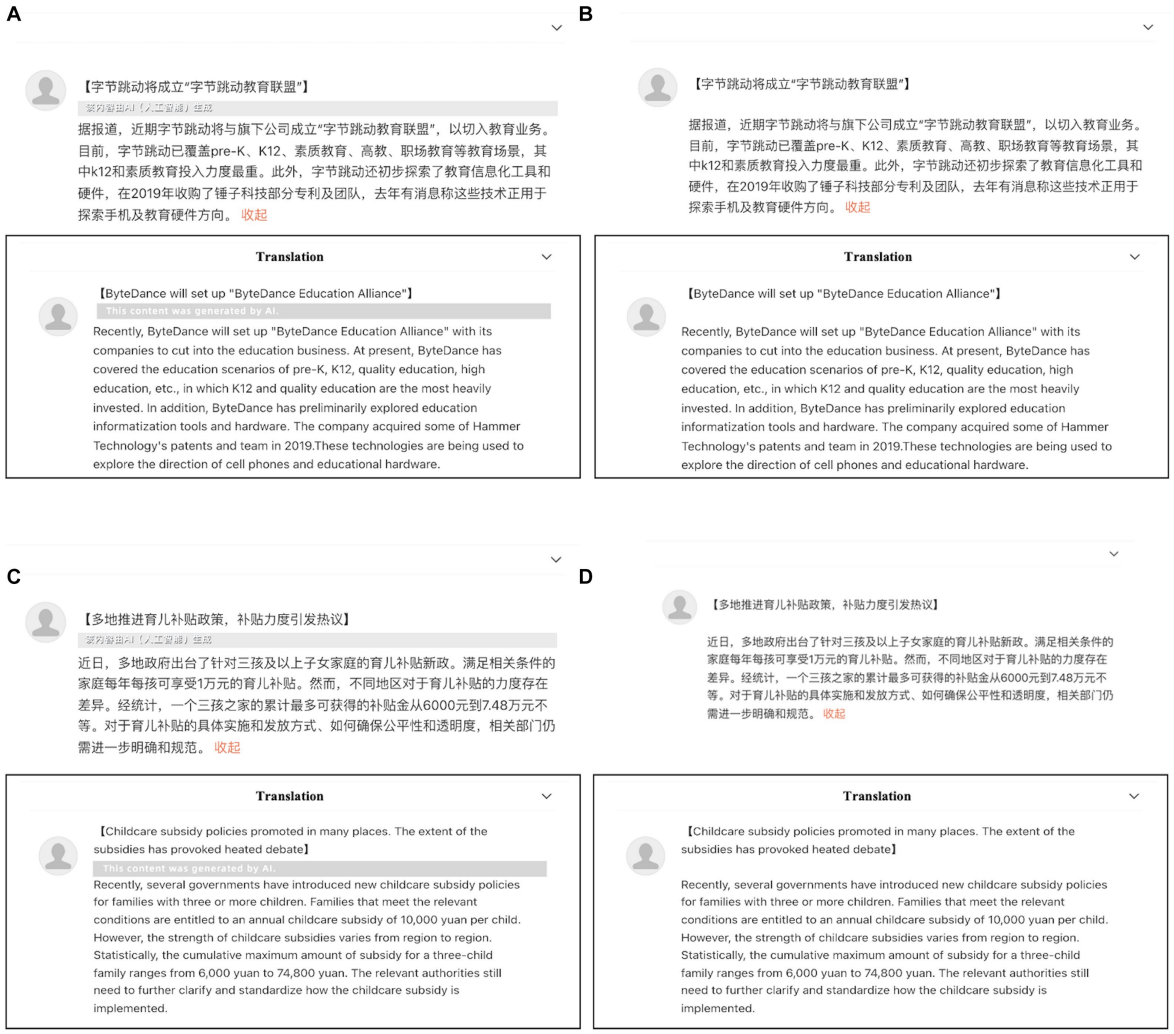
Screenshots of four experimental conditions. **(A)** Descriptive news with AIGC labeling (condition LD). **(B)** Descriptive news without AIGC labeling (condition ND). **(C)** Evaluative news with AIGC labeling (condition LE). **(D)** Evaluative news without AIGC labeling (condition NE).

All automated news reading materials were placed in a presentation interface similar to that of short news tweets on the *Weibo* platform. All short news tweets were presented in the form of pictures, which were made by Photoshop with a resolution (of a photo) of 1,600 × 900 (Materials presented under different experimental conditions are shown in Figure 1).

**Figure 2:**
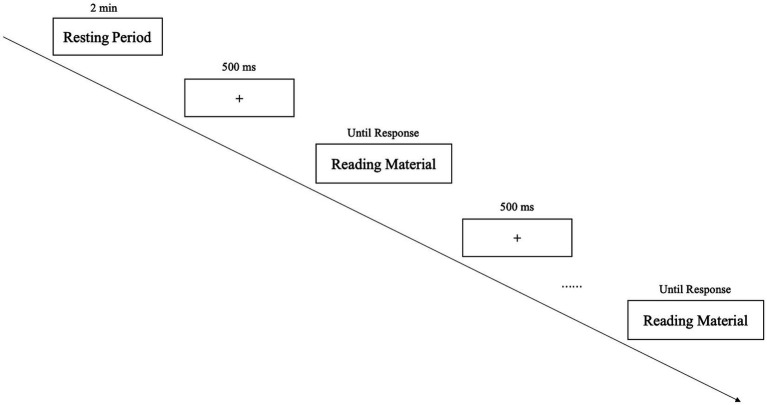
Experimental procedure.

At the beginning of the experiment, participants entered a bright, quiet, and closed laboratory room, isolated from external electromagnetic signals and noise, and sat in a chair 50 cm away from a desktop computer. They were told that they needed to complete news reading and evaluating tasks on the desktop computer. The experimental tasks were presented through E-Prime 3.0. There were four practice trials before the formal experiment and 80 formal trials in the formal experiment, which were presented in the form of four blocks. The sequence of materials presented in each block was random. In each trial, an empty screen was first presented for 500 ms, and then automated news reading material pictures were presented individually. After reading each item of news, participants were asked to rate the content’s trustworthiness by pressing yes/no buttons. When participants hit yes, it meant they thought the content was trustworthy, and when they selected no, it suggested they thought the content was untrustworthy. Then, an empty screen was presented for 500 ms before entering the next trial (The trial procedure is shown in Figure 2). Participants were given 1 min of rest time between the two blocks to ensure that they completed the experiment peacefully. During the experiment, the EEG device continuously recorded participants’ electrical brain signals. After completing the experimental task, participants were also asked to complete a questionnaire about manipulation checks and attention tests. Then, the experiment ended.

### 3.4 Data recordings and analysis

The behavioral data were collected by E-Prime 3.0 and were analyzed by E-Data 3.0 and SPSS 26.0 to calculate individuals’ trustworthiness scores of the whole 20 reading materials. Trustworthiness score was defined as the total number of Yes button responses to the 20 items. A higher score indicates a higher number of articles that participants found trustworthy, which can evaluate participants’ perceived trustworthiness level of the whole reading material.

EEG data were recorded by a Cognionics Quick-30 (CGX, San Diego, CA, USA) 32-channel wireless dry electrode electroencephalograph with channel locations arranged in a 10–20 system. The EEG data sampling rate was 1,000 Hz, and the DC recording, forehead grounding, and recording bandwidth were 0–100 Hz. The left mastoid was used as the reference electrode and was converted to the average reference value of the bilateral mastoid for offline analysis. EEG data were analyzed using EEGLAB 2023. The EEG signals were bandpass filtered at 1–30 Hz, then the EEG signals with large drifts were manually removed, and artifacts, such as blinking, eye movement, and head movement, were removed using independent component analysis (ICA). After obtaining clean data, the data of nine electrode points, F3, Fz, F4, C3, Cz, C4, P3, Pz, and P4, were selected for offline analysis. Fast Fourier transform (FFT) (Hanning window function, 1-width and 50% overlapping) was used to extract the power spectral density (PSD) of the delta (1–4 Hz), theta (4–8 Hz), alpha (8–13 Hz), and beta (13–30 Hz) bands at nine channels. The EEG data used for the analysis were derived from EEG data during the participants’ reading, subtracted from EEG data resting before the experimental task to correct for baseline. In addition, the theta/alpha ratio (TAR), theta/beta ratio (TBR), and frontal EEG asymmetry (FEA) were calculated. For normalization, the natural logarithm of these PSD values was taken, and the average of the PSD for three scalp regions (frontal, central, and parietal) was calculated. The calculation method of FEA comes from Allen, Coan, and Nazarian: first, the alpha frequency (8–13 Hz) power values of electrodes F3 and F4 in the frontal region were taken, and then the formula FEA = LnF4-LnF3 was used (Zhang and Zhou, 2010) to obtain the FEA data. SPSS 26.0 was used for statistical analysis of these data.

## 4 Results

### 4.1 Manipulation check

For the AIGC labeling manipulation check, participants were asked to answer the question at the end of each block: Did you see AIGC labeling while reading? All participants noticed the appearance and absence of AIGC labeling.

For the news type manipulation check, participants were asked to score the degree of fact description and opinion commentary of news materials with a 7-point Likert scale after the experiment. The results showed that there were significant differences between the fact description score (descriptive news, *M* = 4.25, *SD* = 0.75; evaluative news, *M* = 2.91, *SD* = 0.97) and the opinion commentary score (descriptive news, *M* = 2.84, *SD* = 0.88; evaluative news, *M* = 4.30, *SD* = 0.70) [*t*(31) = 5.946, *p* < 0.001, Cohen’s *d* = 1.55; *t*(31) = −6.462, *p* < 0.001, Cohen’s *d* = 1.84] for the two news types.

### 4.2 Trustworthiness score of Reading material

The results of participants’ trustworthiness scores under different experimental conditions are shown in Table 1. Two-way repeated measure ANOVA was used to analyze participants’ button response data. AIGC labeling cues and news types were both within-subject variables. The main effect of labeling cues was significant [*F*(3,96) = 18.489, *p <* 0.001, *η^2^_p_* = 0.374], and the trustworthiness score was lower when AIGC labeling was disclosed, which means that AIGC labeling nudged participants to decrease their content trustworthiness evaluations. The main effect of news type was not significant [*F*(3,96) = 2.884, *p* = 0.099, *η^2^_p_* = 0.085]. The interaction effect between labeling cues and news type was not significant [*F*(3,96) = 0.006, *p* = 0.940, *η^2^_p_* = 0.001], indicating that the nudging effect of AIGC labeling was not affected by news type. Therefore, H1 was supported, but H2 and H3 were rejected.

**Table 1:** Trustworthiness score of reading material in four experimental conditions (*M* ± *SD*).

	Without AIGC labeling	With AIGC labeling
Descriptive news	15.56 ± 2.45	14.27 ± 2.90
Evaluative news	15.00 ± 3.08	13.66 ± 3.10

### 4.3 EEG power spectral density and other EEG indexes

**Figure 3:**
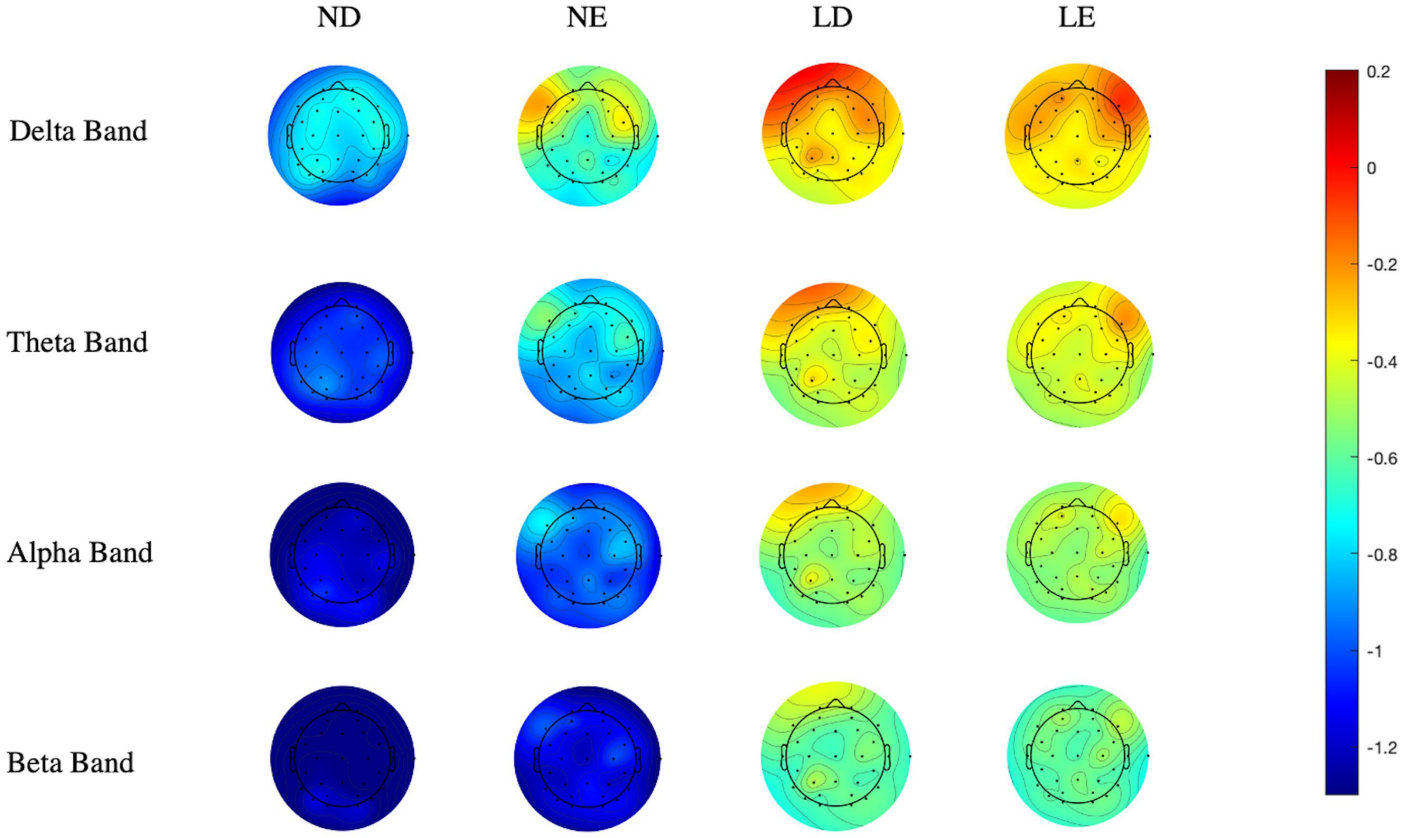
Scalp topographic maps of power spectral density in each frequency band of four experimental conditions (μV2/Hz). ND, descriptive news without AIGC labeling; NE, evaluative news without AIGC labeling; LD, descriptive news with AIGC labeling; LE, evaluative news with AIGC labeling.

**Figure 4:**
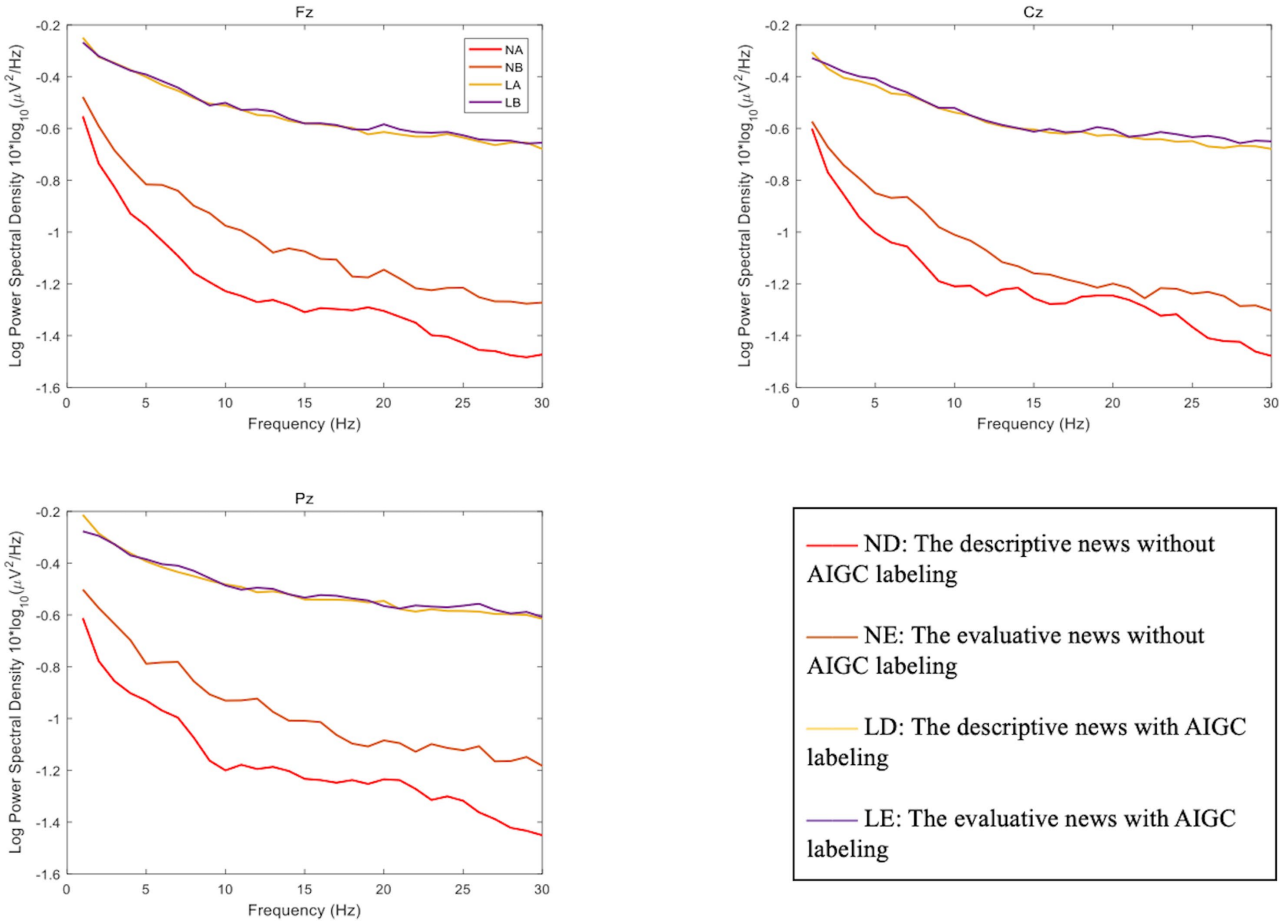
Power spectral density of each frequency band (0–30 Hz) (μV2/Hz). ND, descriptive news without AIGC labeling; NE, evaluative news without AIGC labeling; LD, descriptive news with AIGC labeling; LE, evaluative news with AIGC labeling.

The power spectral density (PSD) of each experimental condition is shown in Table 2, and the scalp topographic maps of the PSD of each frequency band are shown in Figure 3. The line graph of the power spectral density of each frequency band (0–30 Hz) is shown in Figure 4. The three-way repeated measure ANOVA results show the following:

**Table 2:** Power spectral density of each frequency band of participants in four experimental conditions (μV2/Hz) (*M* ± *SD*).

		Without AIGC labeling		With AIGC labeling	
		Descriptive news	Evaluative news	Descriptive news	Evaluative news
Delta band (1–4 Hz)	Frontal	−3.07 ± 0.18	−3.38 ± 0.13	−3.06 ± 0.32	−3.15 ± 0.29
	Central	−3.51 ± 0.10	−3.64 ± 0.20	−3.58 ± 0.40	−3.42 ± 0.40
	Parietal	−3.65 ± 0.28	−3.70 ± 0.25	−3.66 ± 0.14	−3.61 ± 0.48
Theta band (4–8 Hz)	Frontal	−4.18 ± 0.25	−4.50 ± 0.20	−4.28 ± 0.35	−4.39 ± 0.28
	Central	−4.62 ± 0.19	−4.70 ± 0.26	−4.64 ± 0.33	−4.58 ± 0.24
	Parietal	−4.71 ± 0.12	−4.76 ± 0.20	−4.75 ± 0.25	−4. 65 ± 0.19
Alpha band (8–13 Hz)	Frontal	−4.97 ± 0.33	−5.26 ± 0.24	−5.08 ± 0.33	−5.17 ± 0.23
	Central	−5.28 ± 0.25	−5.35 ± 0.31	−5.29 ± 0.39	−5.23 ± 0.26
	Parietal	−5.16 ± 0.18	−5.22 ± 0.24	−5.22 ± 0.37	−5.12 ± 0.22
Beta band (13–30 Hz)	Frontal	−5.77 ± 0.34	−6.01 ± 0.35	−5.90 ± 0.41	−5.87 ± 0.27
	Central	−6.13 ± 0.14	−6.20 ± 0.22	−6.14 ± 0.28	−6.07 ± 0.10
	Parietal	−6.09 ± 0.13	−6.13 ± 0.22	−6.14 ± 0.19	−6.02 ± 0.18

#### 4.3.1 Delta band

The main effect of labeling cues was significant [*F*(3,96) = 17.590, *p* = 0.001, *η^2^_p_* = 0.540], and the delta PSD on automated news with AIGC labeling was stronger than that without AIGC labeling. The main effect of channel location was significant [*F*(3,96) = 32.481, *p <* 0.001, *η^2^_p_* = 0.684], and further post-hoc tests revealed significant differences between frontal and central regions (*p* < 0.001) and between frontal and parietal regions (*p* < 0.001). The interaction effect of news types × channel location was significant [*F*(3,96) = 19.614, *p <* 0.001, *η^2^_p_* = 0.567], and further simple effect analysis revealed significant differences in the frontal region between descriptive news and evaluative news (*p* < 0.05). Other main effects and interaction effects were not significant.

#### 4.3.2 Theta band

The main effect of channel location was significant [*F*(3,96) = 61.980, *p <* 0.001, *η^2^_p_* = 0.805], and further *post hoc* tests revealed significant differences between frontal and central regions (*p* < 0.001) and between frontal and parietal regions (*p* < 0.001). The interaction effect of labeling cues × news type was significant [*F*(3,96) = 4.683, *p* = 0.047, *η^2^_p_* = 0.238], and further simple effect analysis revealed that under the descriptive news condition, the theta PSD with AIGC labeling was significantly stronger than that without AIGC labeling (*p* < 0.001). The interaction effect of news types × channel location was significant [*F*(3,96) = 36.537, *p <* 0.001, *η^2^_p_* = 0.709], and further simple effect analysis revealed significant differences in the frontal region between descriptive news and evaluative news (*p* < 0.001). Other main effects and interaction effects were not significant.

#### 4.3.3 Alpha band

The main effect of channel location was significant [*F*(3,96) = 7.136, *p* = 0.003, *η^2^_p_* = 0.322], and further *post hoc* tests revealed significant differences between frontal and central regions (*p* < 0.05) and between central and parietal regions (*p* < 0.05). The interaction effect of labeling cues × news type was significant [*F*(3,96) = 5.696, *p* = 0.031, *η^2^_p_* = 0.275], and further simple effect analysis revealed that under the descriptive news condition, the alpha PSD with AIGC labeling was significantly stronger than that without AIGC labeling (*p* < 0.05). The interaction effect of news types × channel location was significant [*F*(3,96) = 39.082, *p <* 0.001, *η^2^_p_* = 0.723], and further simple effect analysis revealed significant differences in the frontal region between descriptive news and evaluative news (*p* < 0.001). Other main effects and interaction effects were not significant.

#### 4.3.4 Beta band

The main effect of labeling cues was significant [*F*(3,96) = 14.088, *p* = 0.002, *η^2^_p_* = 0.484], and the beta PSD on automated news with AIGC labeling was stronger than that without AIGC labeling. The main effect of channel location was significant [*F*(3,96) = 712.554, *p* = 0.003, *η^2^_p_* = 0.456], and further *post hoc* tests revealed significant differences between frontal and central regions (*p* < 0.001) and between frontal and parietal regions (*p* < 0.05). The interaction effect of labeling cues × news type was significant [*F*(3,96) = 5.354, *p* = 0.035, *η^2^_p_* = 0.263], and further simple effect analysis revealed that under the descriptive news condition, the beta PSD with AIGC labeling was significantly stronger than that without AIGC labeling cues (*p* < 0.05). The interaction effect of news types × channel location was significant [*F*(3,96) = 25.336, *p <* 0.001, *η^2^_p_* = 0.628], and further simple effect analysis revealed significant differences in the frontal and parietal regions between descriptive news and evaluative news (*p* < 0.05). Other main effects and interaction effects were not significant.

The results of the FEA, TAR, and TBR of each experimental condition are shown in Table 3. The two-way repeated measure ANOVA results show the following:

**Table 3:** FEA, TAR, and TBR in four experimental conditions (*M ± SD*).

	Without AIGC labeling		With AIGC labeling	
	Descriptive news	Evaluative news	Descriptive news	Evaluative news
FEA	−0.09 ± 0.35	−0.03 ± 0.22	−0.15 ± 0.87	−0.10 ± 0.28
TAR	0.78 ± 0.03	0.82 ± 0.03	0.80 ± 0.03	0.81 ± 0.04
TBR	0.66 ± 0.04	0.69 ± 0.06	0.64 ± 0.05	0.70 ± 0.06

FEA, frontal EEG asymmetry; TAR, theta/alpha ratio; TBR, theta/beta ratio.

#### 4.3.5 Frontal EEG asymmetry (FEA)

The main effect of labeling cues was not significant [*F*(3,96) = 0.303, *p* = 0.590, *η^2^_p_* = 0.020]. The main effect of news type was not significant [*F*(3,96) = 0.367, *p* = 0.554, *η^2^_p_* = 0.024]. The interaction effect of labeling cues × news type was not significant [*F*(3,96) = 0.002, *p* = 0.962, *η^2^_p_* < 0.001].

#### 4.3.6 Theta/alpha ratio (TAR)

The main effect of news type was significant [*F*(3,96) = 20.219, *p <* 0.001, *η^2^_p_* = 0.574]. The interaction effect of labeling cues × news type was significant [*F*(3,96) = 7.739, *p* = 0.014, *η^2^_p_* = 0.340], and further simple effect analysis revealed that under the descriptive news condition, the TAR with AIGC labeling was significantly higher than that without AIGC labeling cues (*p* < 0.001).

#### 4.3.7 Theta/beta ratio (TBR)

The main effect of news type was significant [*F*(3,96) = 34.997, *p <* 0.001, *η^2^_p_* = 0.700]. The main effect of labeling cues was not significant [*F*(3,96) = 0.473, *p* = 0.502, *η^2^_p_* = 0.031]. The interaction effect of labeling cues × news type was not significant [*F*(3,96) = 3.852, *p* = 0.069, *η^2^_p_* = 0.204].

## 5 Discussion

Based on the nudge theory and the social background of AI’s deep involvement in news production, the present study focused on the effect of AI authorship disclosure cues on users’ perceptions of automated news. Two disclosure contexts (with AIGC labeling vs. without AIGC labeling) and two news types (descriptive news vs. evaluative news) were used to investigate changes in users’ trustworthiness perceptions. Behavioral results showed that disclosure of AIGC labeling significantly reduced users’ perceived trustworthiness of automated news for both fact-based descriptive and opinion-based evaluative news. Therefore, H1 was supported, while H2 and H3 were not. EEG results showed that the main effect of labeling cues was significant in the delta band and beta band, and the delta PSD and beta PSD with disclosure of AIGC labeling were significantly higher than those without AIGC labeling. In the theta and alpha bands, the interaction effect of labeling cues × news type was significant. Under the condition of descriptive news, both theta PSD and alpha PSD were significantly stronger with AIGC labeling. In addition, the interaction effect of labeling cues × news type was also significant in the TAR index. In descriptive news conditions, TAR with AIGC labeling was higher than that without AIGC labeling. In this study, no influence was found on FEA and TBR.

More specifically, on the behavioral side, the current study revealed that AIGC labeling as a means of increasing news transparency has a significant nudging effect, which can reduce users’ perceived trustworthiness of automated news. This is consistent with previous findings on the effects of transparency cues in news exposure (Weinreich et al., 2008). As a kind of transparency cue in news exposure, labeling can potentially clarify the implied risk information of news and activate users’ critical thinking and cognitive reflection to complete subsequent cognitive processing activities (Otis, 2022). AIGC labeling, as a means of transparency disclosure in journalism, can effectively emphasize the authorship of AI machines and remind users of possible quality risks. Therefore, users are easily nudged by AIGC labeling to stop the mental shortcut of quickly skimming news and reassessing news quality through machine heuristic paths.

Surprisingly, the behavioral results of this study found that the nudging effect of AIGC labeling was not affected by news type, which is inconsistent with the two machine-heuristic pathways proposed by Sundar and Kim (2019) and the results of existing studies on the trustworthiness evaluations of machine writing (Longoni and Cian, 2020). Previous research has supported Sundar and Kim’s view that machine creators are inferior to human authors in terms of subjective judgment, explanatory reasoning, and emotional empathy but are more objective and accurate than human authors (Sundar and Kim, 2019). People were more likely to trust machine-written descriptive essays and be skeptical of machine-written evaluative essays. However, the current study did not find an interaction effect between the labeling cues and news type. When AIGC labeling was presented, participants’ perceived trustworthiness of both news types was significantly reduced. This may be because participants in this study were mostly young people with backgrounds in humanities and social sciences, and their algorithm aversion was relatively high (Dietvorst et al., 2015). This means that regardless of how well machine writers perform or even if they outperform humans in many tasks, participants prefer to interact with human agents. Therefore, in the case of AIGC labeling, participants’ perceived content trustworthiness was lower. However, further research is needed to determine whether algorithm aversive emotion plays a mediating effect.

In terms of EEG, the data revealed that both labeling cues and news type had significant effects on the brain activities of participants. First, the current study found that in the delta band, the delta PSD of participants shown AIGC labeling was significantly higher than that of participants not shown AIGC labeling. Existing studies have claimed that the delta band is the brain wave active during unconscious and deep sleep states and can reflect the fatigue degree of cognitive processing. When the fatigue degree was increased, the delta PSD increased (Lal and Craig, 2001). Therefore, the results of this study may imply that the presence of AIGC labeling cues increases participants’ cognitive fatigue. This may indicate that the authorship disclosure cues did affect users’ information processing by initiating cognitive *System II*. Kahneman and Tversky (1972) proposed the two cognitive operations in the dual processing model: *System I* processing was intuitive, automatic, fast, unconscious, and based on experiences. *System II* processing was rational, controlled, slow, conscious, and based on consequences. Because AIGC labeling might activate participants’ analytical minds and critical thinking models, they perceived more fatigue in the presence of the labeling cue. This supported the theoretical assumptions of educative nudges. The so-called educative nudges attempted to strengthen the hand of *System II* by improving the role of deliberation and people’s considered judgments, including content labels, warnings, and reminders (Hertwig and Grüne-Yanoff, 2017; Pennycook et al., 2020; Sunstein, 2021; Pennycook and Rand, 2022). AIGC labeling may create cognitive friction during participants’ information processing. Participants were nudged by AIGC labeling to slow the pace of evaluation and reinvest limited cognitive resources in assessing the quality of information, which led to in-depth cognitive processing. Second, the present study found that in the theta and alpha bands, participants reading descriptive news with AIGC labeling had significantly higher theta PSD and alpha PSD than those reading descriptive news without AIGC labeling. In the evaluative news, theta PSD and alpha PSD were also higher with the labeling cues, but the difference was not significant compared with the absence of the labeling cues. Existing studies have suggested that the enhancement of theta PSD reflects the improvement of cognitive control in the brain (Gruzelier, 2014), while the enhancement of alpha PSD is associated with higher emotional arousal and cognitive complexity (Borghini et al., 2014). These results implied that AIGC labeling can increase users’ cognitive agency and short-term cognitive competence, such as evoking stronger cognitive activities, attracting them to devote more cognitive resources to processing information, inhibiting other cognitive activities unrelated to information processing, and enhancing the depth of cognitive processing. This was also correlated with the cognitive fatigue results reflected by the delta PSD, indicating that the labeling cues can enhance participants’ cognitive processing degree. The results also showed that this AIGC labeling effect was stronger for the descriptive news than the evaluative news. On the one hand, this might be due to users’ motivation to utilize news aggregation platforms. Lee and Chyi (2015) found that information-seeking motivation was a statistically significant predictor for news aggregator use. News aggregation platform users were more likely to verify the authorship of objective information. Only if the author was credibly verified would the user consider the information provided to be sufficiently objective and credible. In contrast, users did not care much about the authorship of viewpoint information in news aggregation platforms. The degree of cognitive processing arousal was more influenced by the consistency between the viewpoint and users’ established values (Choi and Lim, 2019). Thus, AIGC labeling, as an authorship disclosure cue, was more likely to trigger larger effects in descriptive news. On the other hand, this was also due to the different reading habits of the two news types. Some researchers empirically showed that users more thoroughly verify descriptive news than evaluative news (Flanagin and Metzger, 2000). Users would more stringently verify factual descriptive news, which deals with information that significantly impacts their lives (notices, announcements, for example) than opinion assessment information, involving evaluations, debates, and predictions. Hence, the degree of cognitive control and cognitive arousal induced by descriptive news might be higher than that induced by evaluative news. Third, in the beta band, the current study also found that the beta PSD with AIGC labeling was significantly higher than that without AIGC labeling. In previous studies, attention concentration and high-intensity cognitive activities have been shown to cause a higher degree of beta PSD (Fernández et al., 1995). This indicated that AIGC labeling may also improve users’ attention concentration and activate high-intensity cognitive processing, which means that AIGC labeling not only impacts the deep cognitive process (i.e., reasoning and judgment) but also significantly impacts the superficial attention process. This becomes strong evidence supporting a limited-attention utility model that is based on a theory about inattention to accuracy on social media proposed by Pennycook and Rand (2022). They claimed that users do not lack the ability to process and judge information, but they often have cognitive limitations because of the wrong attention attracted by social media. Therefore, they often cannot distinguish information quality and fall into the whirlpool of fake news. Pennycook and Rand (2022) proposed that to improve users’ information distinguishing ability, it is necessary to attract users’ attention through accuracy cues. Undoubtedly, AIGC labeling, as a cue of authorship disclosure, can attract users’ attention to re-evaluate information quality. It is an effective educative nudge, especially in increasing users’ short-term cognitive motivation and cognitive skills.

In addition, there were some interesting findings on other EEG indexes. First, FEA was negative in all experimental conditions and lower when AIGC labeling was present. FEA is usually used to measure the convergence and avoidance of cognitive motivations. When FEA was positive, the left frontal cortex was more active than the right frontal cortex, indicating that individuals show cognitive motivation proximity and tend to show the converging action orientation. When FEA was negative, the right frontal cortex was more active than the left, and individuals tended to show avoidance of cognitive motivation (Coan and Allen, 2004; Shokri and Nosratabadi, 2021). The FEA results in the present study indicated that all participants had a slight avoidance motivation for this news reading task. When the news was labeled as generated by AI, participants’ potential avoidance motivations were strengthened. Second, it was found that the TAR of descriptive news was substantially higher than that of evaluative news, and there was a significant difference between the two news types when AIGC labeling appeared. In existing studies, TAR was usually an index of cognitive load. The larger the cognitive load is, the larger the TAR is (Yu et al., 2021). Therefore, the results indicated that the cognitive loads of participants were different when reading the two kinds of news, which reflected the effectiveness of news type manipulation. It also showed that the labeling cue can increase the cognitive load difference between the two news types, as evidenced by reducing the cognitive load of the evaluating news and increasing the cognitive load of the descriptive news.

In conclusion, from the perspective of cognitive neuroscience, the present study explored the impact of machine authorship labeling on users’ content perceptions more microscopically. Notably, the behavioral results of this study can be correlated with the EEG results. AIGC labeling cues can reduce users’ perceived trustworthiness of related content, and the decrease results from users’ deeper information processing nudged by AIGC labeling. The nudging effect of AIGC labeling is manifested by providing hints about the potential information risks, and users are encouraged to shift their limited attention and cognitive resources to re-evaluate the quality of information to obtain more prudent judgment results. The theoretical significance of this study is that combining EEG technology and behavioral experiment methods can supplement the theoretical perspective on transparent disclosure nudging in the internet content governance research field and confirm the efficacy of transparent disclosure via authorship labeling cues. In a practical sense, the present study can offer media organizations guidance on how to utilize AIGC labeling and provide a view of how content is governed by content labeling, thereby helping to regulate and direct the media industry landscape in the face of AI’s pervasive presence.

The current study also had the following limitations. First, limited by funds and energy, the present study considered only two representative news types, and the news was mainly presented in text. In future, richer news types, such as pictures combined with texts, should be investigated more comprehensively. Second, although we tried our best to avoid the disruptive effect of gender and professional background, we could not include students from all disciplinary backgrounds, which may have allowed some participants with higher algorithm aversion to be recruited. Finally, although the present research used the most advanced generative AI technology on the market to generate experimental reading materials, AI technology still cannot produce content that is similar to human writing, which means that subtle errors in experimental results are unavoidable. With the development of AI technology in future, this problem is expected to be effectively solved.

## Data availability statement

The raw data supporting the conclusions of this article will be made available by the authors, without undue reservation.

## Ethics statement

The studies involving humans were approved by Ethics Committee at the School of Journalism and Communication, Beijing Normal University. The studies were conducted in accordance with the local legislation and institutional requirements. The participants provided their written informed consent to participate in this study.

## Author contributions

YL: Conceptualization, Data curation, Methodology, Writing – original draft, Writing – review & editing. SW: Data curation, Writing – review & editing, Methodology. GY: Conceptualization, Funding acquisition, Resources, Writing – review & editing, Project administration, Supervision.
